# Characterization of the Involvement of Tumour Necrosis Factor (TNF)-α-Stimulated Gene 6 (TSG-6) in Ischemic Brain Injury Caused by Middle Cerebral Artery Occlusion in Mouse

**DOI:** 10.3390/ijms24065800

**Published:** 2023-03-18

**Authors:** Chiara Di Santo, Daniele La Russa, Rosaria Greco, Alessandra Persico, Anna Maria Zanaboni, Giacinto Bagetta, Diana Amantea

**Affiliations:** 1 Section of Preclinical and Translational Pharmacology, Department of Pharmacy, Health and Nutritional Sciences, University of Calabria, 87036 Rende, CS, Italy; 2 IRCCS Mondino Foundation, Via Mondino 2, 27100 Pavia, PV, Italy

**Keywords:** cerebral ischemia, immune system, neurodegeneration, neuroinflammation, stroke, TSG-6

## Abstract

The identification of novel targets to modulate the immune response triggered by cerebral ischemia is crucial to promote the development of effective stroke therapeutics. Since tumour necrosis factor (TNF)-α-stimulated gene 6 (TSG-6), a hyaluronate (HA)-binding protein, is involved in the regulation of immune and stromal cell functions in acute neurodegeneration, we aimed to characterize its involvement in ischemic stroke. Transient middle cerebral artery occlusion (1 h MCAo, followed by 6 to 48 of reperfusion) in mice resulted in a significant elevation in cerebral TSG-6 protein levels, mainly localized in neurons and myeloid cells of the lesioned hemisphere. These myeloid cells were clearly infiltrating from the blood, strongly suggesting that brain ischemia also affects TSG-6 in the periphery. Accordingly, *TSG-6* mRNA expression was elevated in peripheral blood mononuclear cells (PBMCs) from patients 48 h after ischemic stroke onset, and TSG-6 protein expression was higher in the plasma of mice subjected to 1 h MCAo followed by 48 h of reperfusion. Surprisingly, plasma TSG-6 levels were reduced in the acute phase (i.e., within 24 h of reperfusion) when compared to sham-operated mice, supporting the hypothesis of a detrimental role of TSG-6 in the early reperfusion stage. Accordingly, systemic acute administration of recombinant mouse TSG-6 increased brain levels of the M2 marker Ym1, providing a significant reduction in the brain infarct volume and general neurological deficits in mice subjected to transient MCAo. These findings suggest a pivotal role of TSG-6 in ischemic stroke pathobiology and underscore the clinical relevance of further investigating the mechanisms underlying its immunoregulatory role.

## 1. Introduction

Cerebral ischemia triggered by stroke is a major cause of mortality and long-term disability, generating a global healthcare burden that is expected to grow if prevention and treatment strategies are not implemented in the near future [1]. In fact, currently approved therapies only rely on reperfusion of the lesioned tissue by pharmacological or mechanical thrombus lysis/removal [2,3,4,5], generating high expectations for the development of novel neuroprotective strategies. However, despite the great efforts made in recent decades to characterize the pathobiological mechanisms implicated in ischemic brain injury, to date, none of the identified putative targets have been successfully translated into effective therapies [6,7,8].

The immune system exerts a dualistic role in the progression of ischemic brain damage, playing detrimental or protective/regenerative roles depending on the specific soluble mediators (e.g., pro- or anti-inflammatory cytokines) or cellular phenotype (e.g., inflammatory M1 or reparative M2) involved [9,10,11]. Indeed, recent studies have validated the suitability of a rational immunomodulation by targeting central and/or peripheral immune responses involved in ischemic pathobiology [9,10,12,13]. Thus, the identification of novel targets to modulate the immune responses triggered by cerebral ischemia represents a desirable aim with strong translational value.

Recent work has highlighted that the multifunctional protein tumour necrosis factor (TNF)-α-stimulated gene 6 (TSG-6) may play a role in acute neurodegenerative diseases [14]. TSG-6 is a member of the family of hyaluronate (HA) binding proteins, involved in cell–cell and cell–matrix interactions during inflammation and tumorigenesis [15,16,17]. TSG-6 is constitutively expressed in the brain, while its upregulation occurs upon inflammatory stimuli in a wide variety of cell types, including astrocytes, monocytes/macrophages, dendritic cells, mesenchymal stem/stromal cells (MSCs), vascular smooth muscle cells (VSMCs) and fibroblasts [18,19,20,21,22]. Among its various activities, TSG-6 regulates immune and stromal cells functions and affects extracellular matrix assembly and interaction with cell surface receptors and soluble mediators (e.g., chemokines) [23], exerting anti-inflammatory and immunosuppressive effects in diverse pathological contexts, including neuroinflammation [24,25,26,27,28,29,30,31]. Accordingly, this protein protects tissues from the injurious effects caused by spinal cord injuries [32] and, most notably, acute cerebral injuries [33,34,35,36,37,38]. Notably, TSG-6 expression is elevated in the peri-infarct and infarcted brain tissue of stroke patients [39] and in the plasma of non-cardioembolic acute ischemic stroke patients, where it is positively associated with disease severity and lesion volume [40]. In rodent models of global and focal cerebral ischemia, TSG-6 mediates the protective effects conferred by systemic administration of MSCs [37,41,42], although its direct effects and endogenous functions have not been fully clarified yet. Thus, here we aim to characterize the involvement of central and peripheral TSG-6 in ischemic brain injury.

## 2. Results

### 2.1. Analysis of the Cerebral Expression of TSG-6 following Transient MCAo in Mice

**Figure 1:**
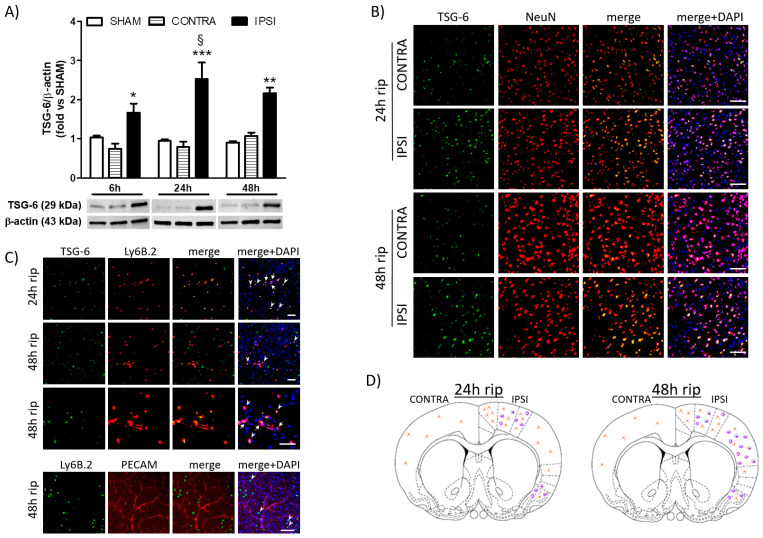
Tumour necrosis factor (TNF)-α-stimulated gene 6 (TSG-6) protein expression is elevated in the brain of mice subjected to transient MCAo. (**A**) TSG-6 protein expression levels detected by Western blotting in the ipsilateral (ischemic and IPSI) and contralateral (CONTRA) cortex of mice subjected to 1 h MCAo followed by 6, 24 or 48 h of reperfusion or mice subjected to sham surgery (SHAM). Data are expressed as means ± S.E.M.; * *p* < 0.05 vs. CONTRA 6 h, *** *p* < 0.001 vs. SHAM 24 h and vs. CONTRA 24 h, § *p* < 0.05 vs. IPSI 6 h, ** *p* < 0.01 vs. SHAM 48 h and vs. IPSI 48 h (two-way ANOVA followed by Bonferroni post-test; *n* = 6–8 mice per experimental group). (**B**) Representative immunofluorescence images showing colocalization (yellow overlapping in merge panels) of TSG6 (green fluorescence) with NeuN immunopositive neurons (red fluorescence) in the ipsilateral and contralateral cortex of mice subjected to 1 h middle cerebral artery occlusion (MCAo) followed by 24 or 48 h of reperfusion. Nuclei were counterstained with DAPI (blue signal). Scale bar = 150 μm. (**C**) Representative immunofluorescence images showing colocalization (yellow overlapping in merge panels) of TSG6 (green fluorescence) with Ly6B.2 immunopositive myeloid cells (i.e., granulocytes and monocytes/macrophages, red fluorescence) in the ipsilateral cortex of mice subjected to 1 h MCAo followed by 24 or 48 h of reperfusion. The endothelium was labelled with anti-PECAM1 and the nuclei were counterstained with DAPI (blue signal). Arrows and arrowheads indicate TSG-6 immunopositive myeloid cells in perivascular spaces or infiltrating the brain parenchyma, respectively. Scale bar = 75 μm. (**D**) Schematic representation of the regional and cellular distribution of TSG-6 immunoreactivity in the brain of mice subjected to 1 h MCAo followed by 24 or 48 h of reperfusion. Drawings were obtained from the typical distribution observed by microscopic examination of brain sections (0.70 mm anterior to Bregma) processed for immunofluorescence, as described in the methods section. NeuN and Ly6B.2 immunopositive cells that express TSG-6 are represented by orange or purple symbolic drawings, respectively.

Transient cerebral ischemia caused by 1 h MCAo, followed by 6 to 48 h of reperfusion, resulted in a significant elevation of TSG-6 protein levels in the ipsilateral cortex of mice, reaching a peak 24 h after the beginning of reperfusion (Figure 1A). By immunofluorescence analysis, we observed that TSG6 was barely evident in a few NeuN immunopositive neurons of the contralateral hemisphere, and it was more abundantly expressed in neurons of the ipsilateral peri-infarct tissue (including the motor and frontal cortices) after 24 h or 48 h of reperfusion (Figure 1B). Moreover, expression of TSG6 was observed in Ly6B.2 immunopositive myeloid cells (i.e., granulocytes and monocytes/macrophages) populating the penumbral cortex at 24 h and 48 h of reperfusion and more intensely in the core region (i.e., parietal cortex) at 48 h of reperfusion (Figure 1C). A schematic representation of the cellular and regional expression of TSG-6 in mice brains after ischemia-reperfusion injury is reported in Figure 1D. These TSG-6 immunopositive myeloid cells were likely infiltrating from the blood vessels, as shown in Figure 1C (arrows and arrowheads).

### 2.2. Analysis of Blood Expression of TSG-6 after Ischemic Stroke in Mice and Patients

**Figure 2:**
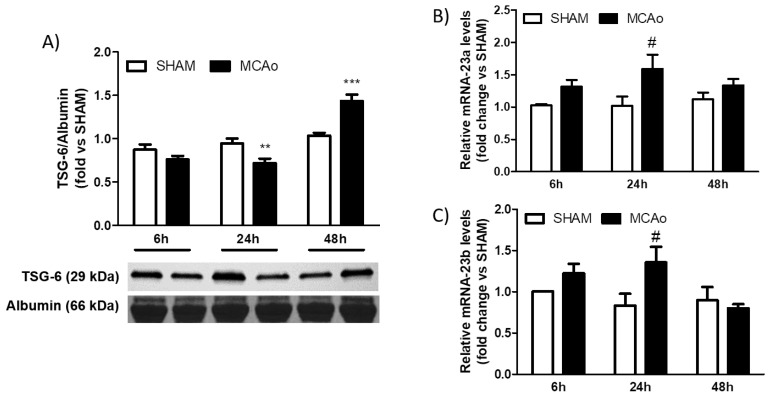
Transient MCAo modulates plasma levels of TSG-6 protein and of miR-23a and miR-23b. (**A**) TSG-6 protein, (**B**) miR-23a or (**C**) miR-23b relative expression levels in plasma of mice following sham surgery (SHAM) or 1 h MCAo (MCAo) followed by 6, 24 or 48 h of reperfusion. Data are expressed as means ± S.E.M.; ** *p* < 0.01 vs. SHAM 24 h, *** *p* < 0.001 vs. SHAM 48 h, # *p* < 0.05 vs. SHAM 24 h (two-way ANOVA followed by Bonferroni post-test, *n* = 5–8 mice per experimental group).

These findings highlight that, in addition to the local (i.e., neuronal) elevation of TSG-6, ischemia affects the expression of this protein in the periphery. Accordingly, we found that TSG-6 protein levels in plasma were acutely (i.e., within 24 h after the beginning of reperfusion) reduced in mice subjected to 1 h MCAo when compared to sham surgery (Figure 2A). By contrast, at later stages (i.e., after 48 h of reperfusion), TSG-6 protein levels were significantly higher than sham (Figure 2A). 

Using the TargetScanMouse database (https://www.targetscan.org/mmu_80/ (accessed on 2 September 2022)), we performed a reverse target prediction analysis to identify miR-23a and miR-23b based on their potential to regulate TSG-6. Interestingly, reduced TSG-6 protein levels observed at 24 h of reperfusion were coincident with higher miR-23a (Figure 2B) and miR-23b (Figure 2C) expression in plasma when compared to sham. Conversely, after 48 h of reperfusion, circulating levels of these miRNAs were not significantly different from sham-operated animals (Figure 2B,C).

**Figure 3:**
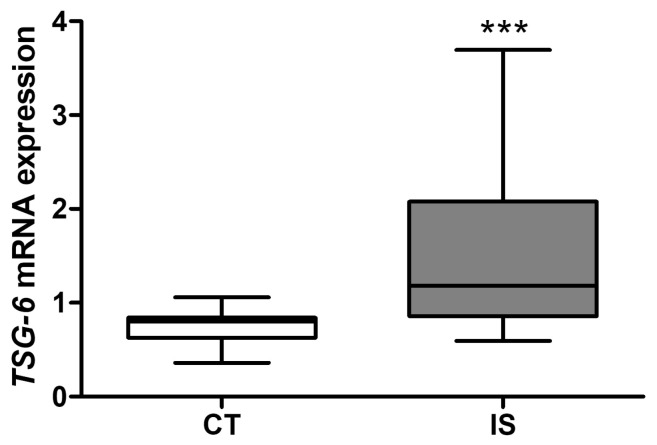
*TSG-6* mRNA expression is elevated 48 h after ischemic stroke in PBMC from patients. *TSG-6* mRNA levels in peripheral blood mononuclear cells (PBMCs) isolated from blood of healthy subjects (CT; *n* = 13) or patients subjected to ischemic stroke 48 h before (IS; *n*= 25). Data are shown as median, interquartile range and minimum and maximum values; *** *p* < 0.01 vs. CT (Mann–Whitney test).

To further characterize TSG-6-mediated responses in blood and to strengthen the clinical relevance of our findings, we explored expression of TSG-6 in circulating peripheral blood mononuclear cells (PBMCs) from ischemic stroke patients. As shown in Figure 3, *TSG-6* mRNA expression was significantly increased in PBMC 48 h after stroke onset, which was coincident with the plasma TSG-6 protein level elevation observed in mice after 48 h of reperfusion (Figure 2A), strengthening the hypothesis that ischemic brain injury affects TSG-6 levels in the periphery.

### 2.3. Neuroprotective Effects of Systemic Administration of TSG-6 in Mice Subjected to MCAo

**Figure 4:**
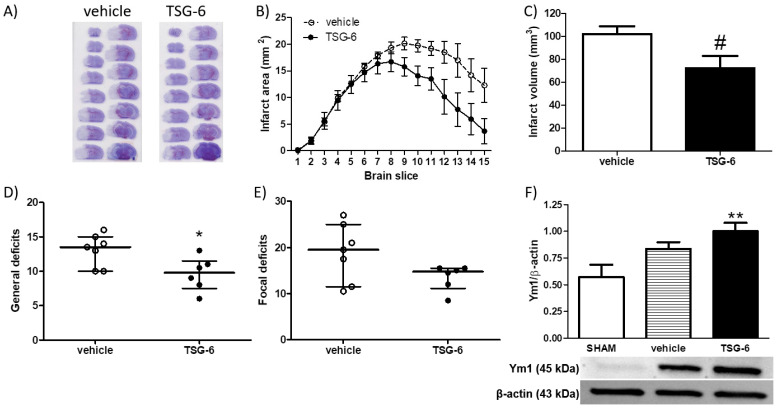
Intravenous acute administration of recombinant TSG-6 significantly reduces brain damage and neurological deficits while promoting cerebral expression of M2-like phenotypes following focal cerebral ischemia in mice. (**A**) Representative cresyl violet-stained coronal brain sections, (**B**) brain infarct area and (**C**) volume in mice subjected to 1 h MCAo followed by 48 h of reperfusion and intravenously injected with 30 μg recombinant mouse TSG-6 or vehicle (100 μL PBS) upon reperfusion. Data are expressed as means ± S.E.M.; # *p* < 0.05 vs. vehicle (unpaired Student’s t test). Effects of the latter treatment on (**D**) general and (**E**) focal neurological deficits caused in mice by 1 h MCAo followed by 48 h of reperfusion. Data are expressed as medians and interquartile ranges; * *p* < 0.05 vs. PBS (two-tailed Mann–Whitney test); *n* = 6–7 mice per experimental group. (**F**) Western blotting analysis of Ym1 protein levels in whole brain homogenates from mice subjected to SHAM surgery or 1 h MCAo followed by 48 h of reperfusion and intravenously injected with TSG-6 or vehicle upon reperfusion. Data are expressed as means ± S.E.M.; ** *p* < 0.01 vs. SHAM (one-way ANOVA followed by Tukey post-test, *n* = 5–7 mice per experimental group).

Based on these findings, we hypothesised that late elevation of TSG-6 levels may represent a compensatory mechanism to counteract damage, whereas acute plasma reduction of TSG-6 may be detrimental, thus contributing to the progression of ischemic brain damage. To clarify this issue, we intravenously (i.v.) administered recombinant mouse TSG-6, at a dose of 30 μg/mouse, selected on the basis of previously published observations [35,43,44]. Thus, TSG-6 suspended in a vehicle (phosphate buffered saline, PBS) was administered (30 μg TSG-6/100 μL PBS/mouse) i.v. upon reperfusion (i.e., after 1 h MCAo) and infarct brain damage and neurological deficits were measured 48 h later. Figure 4 (panels A–C) shows that systemic administration of TSG-6 significantly reduced brain infarct damage caused by 1 h MCAo, whereas the cerebral oedema was not affected. Neuroprotection was also associated with attenuation of general neurological deficits caused by the ischemic insult (Figure 4, panels D and E). Interestingly, neuroprotection by i.v. administration of TSG-6 was associated with a significant elevation of cerebral levels of the M2 marker Ym1 (Figure 4, panel F), strongly supporting the hypothesis that this multifunctional protein exerts an important immunomodulatory function that may underlie its neuroprotective effects against ischemic injury.

## 3. Discussion

The present study originally demonstrates that ischemic stroke injury affects TSG-6 expression levels both in the brain and in the blood. In mice, MCAo resulted in a significant elevation of the neuronal expression of TSG-6 protein in the lesioned hemisphere, with the most intense response observed in the peri-ischemic regions up to 48 h after the beginning of reperfusion. Elevated expression of TSG-6 was also associated with Ly6B.2 myeloid cells populating the ischemic penumbra after 24 h of reperfusion or the entire lesioned region (i.e., both the core and the penumbra) after 48 h of reperfusion. Most of these myeloid cells were clearly infiltrating from the blood, strongly suggesting that ischemic brain injury also affects TSG-6 in the periphery. Accordingly, *TSG-6* mRNA expression was elevated in PBMC from ischemic stroke patients 48 h after symptoms onset. In agreement with these findings, TSG-6 protein expression was elevated in the plasma of mice subjected to 1 h MCAo followed by 48 h of reperfusion. However, plasma levels of TSG-6 were surprisingly reduced in the acute phase (i.e., 6 to 24 h after reperfusion) when compared to sham-operated mice. In this context, circulating levels of miR-23a and miR-23b were correlated with plasma TSG-6 levels in the acute phase (i.e., up to 24 h after the beginning of reperfusion), whereas this correlation was lost 48 h after the insult.

Given the protective and anti-inflammatory effects of TSG-6 in other neuropathological contexts, we hypothesized that its reduction during the acute phase might be involved in the detrimental effects of the ischemic insult. Accordingly, systemic acute administration of recombinant mouse TSG-6 elevated brain levels of the M2 marker Ym1 and provided significant neuroprotection by reducing brain infarct volume and general neurological deficits in mice subjected to transient MCAo.

TSG-6 is a secretory hyaluronate (HA)-binding protein, constitutively expressed in various tissues, including the brain and spinal cord. In the developing rat brain, it shows different expression patterns, being involved in oligodendrocyte maturation and neuronal precursor cell migration [45]. Despite the debated expression during embryonic development, TSG-6 has been observed in astrocytes of the mature rat brain and spinal cord [21], contributing to their maturation, since *TSG-6* null mice display a lower density of cerebral GFAP+ astrocytes [21]. Nevertheless, the selective localization of TSG-6 in astrocytes reported by some studies was not confirmed by others that reported a more widespread distribution, also including microglia and neurons, especially under neurodegenerative/inflammatory conditions [23,34,39,46]. Accordingly, we observed that TSG-6 was almost exclusively expressed in a few NeuN immunopositive neurons of the control (i.e., sham or contralateral) brain tissue. However, we detected an increase in TSG-6 protein levels in the ischemic hemisphere, as the signal was located in neurons and also in Ly6B.2 immunopositive myeloid cells (i.e., granulocytes and monocytes/macrophages) populating the penumbral cortex at 24 h and 48 h of reperfusion and more intensely in the core region (i.e., parietal cortex) at 48 h of reperfusion. Our findings are consistent with the evidence showing an upregulation of TSG-6 in the cerebral cortex of rats following global cerebral ischemia [37]. More importantly, our findings are in agreement with the observation that *TSG-6* mRNA and protein levels increase in the peri-infarct and infarcted brain tissue when compared to the contralateral hemisphere of ischemic stroke patients, as protein staining was associated with damaged neurons and inflammatory mononuclear cells 3 to 29 days after the insult [39]. Interestingly, TSG-6 elevation was coincident with increased HA levels in the lesioned brain and in the serum of stroke patients, together with an elevated expression of the HA receptor CD44 in damaged neurons and inflammatory mononuclear cells 3 to 17 days after stroke [39]. Increased HA synthesis and up-regulation of CD44 in microglia, macrophages and microvessels of the ischemic brain tissue were also reported to occur in rodents following focal cerebral ischemia [47,48]. TSG-6 elevation in the ischemic tissue and the preferential synthesis of high molecular weight HA are probably involved in the regulation of inflammatory responses and in tissue remodelling after ischemic stroke [39,47,48].

TSG-6 expression can be induced by a diverse range of inflammatory stimuli (i.e., TNF, IL-1 and LPS) in a wide variety of cell types, such as monocytes/macrophages, dendritic cells, astrocytes, mesenchymal stem/stromal cells (MSCs), vascular smooth muscle cells (VSMCs) and fibroblasts [18,19,20,21,22]. Thus, TSG-6 regulates immune and stromal cell functions and exerts anti-inflammatory and immunosuppressive effects by direct modulation of inflammatory cells or by regulation of the organization/assembly of extracellular HA matrices [23,24,25,26,27,28,29,30,31]. Although the anti-inflammatory and immunoregulatory functions of TSG-6 have been observed in various neurological disorders, including acute brain injury due to trauma, ischemia or haemorrhage [14], the majority of these findings focussed on the role of this protein as mediator of the beneficial effects of MSCs, whereas knowledge of the endogenous or direct functions of TSG-6 is poor. Indeed, upregulation of the cerebral expression of TSG-6 was reported to mediate the protective effects of intravenous administration of MSCs in rats subjected to global cerebral ischemia by attenuating the expression of neutrophil elastase and of the inflammatory cytokines IL-1β, IL-6 and TNF-α in the lesioned brain [37,41]. In addition, TSG-6 was demonstrated to mediate the anti-inflammatory effects of MSCs by inhibiting NF-κB signalling pathways and downstream cerebral inflammatory reactions caused by intracerebral or subarachnoid haemorrhage [33,49] or traumatic brain injury [38,50] in rats.

Modulation of TSG-6 was not only restricted to the ischemic brain, as we observed that protein levels were significantly modulated in the plasma of mice subjected to 1 h MCAo. In particular, plasma TSG-6 protein levels were found to be reduced during the acute phase (i.e., within 24 h of the beginning of reperfusion), while they increased at later reperfusion stages (i.e., after 48 h) when compared to sham-operated mice. The latter finding is consistent with our data showing elevated mRNA expression level of *TSG-6* in PBMC of ischemic stroke patients and with the evidence that non-cardioembolic acute (i.e., within 24 h from symptoms onset) ischemic stroke patients display higher plasma TSG-6 levels than control subjects [40]. In those patients, plasma TSG-6 levels were positively correlated with stroke severity at admission, the lesion volume, the neutrophil count, the neutrophil-to-lymphocyte ratio and interleukin-8 levels. Moreover, increased TSG-6 plasma levels were independently associated with a 3 months poor prognosis, while an elevated TSG-6 to IL-8 ratio predicted a favourable outcome after 3 months [40]. Circulating TSG-6 levels, also reported to be increased in patients with coronary artery disease or carotid stenosis, have been suggested to be derived from endothelial and arterial smooth muscle cells or from monocyte-derived macrophages stimulated by inflammatory mediators [51,52]. The latter evidence is in agreement with our findings demonstrating elevation of *TSG-6* mRNA levels in PBMC from ischemic stroke patients and with the observation that cerebral elevation of TSG-6 depends on its local release by neurons and by blood-borne infiltrating myeloid cells, especially at later reperfusion times. This may also explain the elevation of plasma TSG-6 occurring after 48 h of reperfusion, which might actually represent a compensatory anti-inflammatory mechanism, as also underscored in other inflammatory contexts [45], likely through immunoregulatory effects that promote polarization of myeloid cells towards M2-like protective phenotypes. Conversely, we speculate that the reduced levels of circulating TSG-6 observed in the acute phase after stroke may represent a crucial mechanism implicated in the detrimental acute inflammatory reaction. To verify this hypothesis, we have administered recombinant TSG-6 at the time of reperfusion and we observed that it caused a significant elevation of cerebral levels of the M2 marker Ym1 and a reduction in cerebral lesions and neurological deficits. This is the first evidence of neuroprotection provided by systemic administration of TSG-6 in ischemic stroke; the few other studies available demonstrate its efficacy in other acute neurodegenerative contexts. In fact, intravenous treatment with TSG-6 was reported to decrease neutrophil extravasation, matrix metalloproteinase (MMP)-9 expression and the resulting BBB leakage caused by TBI in mice, thus promoting neurogenesis and attenuating long-term consequences, such as memory impairments and depressive-like behaviour [35]. Moreover, intracerebroventricular (i.c.v.) administration of recombinant TSG-6 in rats subjected to SAH inhibited the microglia shift towards inflammatory phenotypes, attenuated TNF-α expression and upregulated IL-10 expression, thus reducing brain oedema and neurological deficits [34,53]. In turn, knockdown of endogenous *TSG-6* by siRNA elevated the (CD86+) M1 vs. (CD163+) M2 ratio in cerebral microglia and aggravated neurological deficits 24 h after SAH [34,53]. In agreement with these previous findings, we observed significant immunomodulatory and neuroprotective effects of exogenously administered TSG-6. However, in order to shed light on the central vs. peripheral mechanisms implicated in such beneficial effects, further investigation would be necessary to understand the systemic responses elicited by intravenously administered TSG-6.

The immunomodulatory functions of TSG-6 have been widely investigated. Notably, myeloid immune cells produce high levels of TSG-6 in response to inflammatory stimuli [54], whereby TSG-6 has been suggested to act in an autocrine mode on macrophages to promote their transition from inflammatory to anti-inflammatory and immunoregulatory phenotypes [51,55,56,57], likely through suppression of Toll-like receptor (TLR)-4/NF-κB pathways [25,27,56,58,59,60]. Notably, TLR2-related pathways regulate microglia polarization, whereas NF-κB and p38 are implicated in the polarization shift of microglia/macrophages occurring after ischemic stroke [61,62,63,64,65,66]. In fact, BV2 microglia exposed to MSCs before being challenged with LPS reduced their expression of typical early and late M1 markers (iNOS, IL-1β, CD16 and CD86), while elevating M2 polarization markers (CD206 and Arg1) [67,68]. Thus, there is strong evidence to support a pivotal role of TSG-6 in the regulation of M1 to M2 polarization shift of myeloid cells under neuroinflammatory conditions. Our findings strongly suggest that the immunomodulatory functions of TSG-6 may be crucial to its neuroprotective properties in ischemic stroke.

## 4. Materials and Methods

### 4.1. Animals

Adult C57Bl/6J male mice (8–10 weeks old) were purchased from Charles River (Calco, Como, Italy) and were housed under standard environmental conditions (i.e., an ambient temperature of 22 °C, a relative humidity of 65% and 12 h/12 h light/dark cycle), with ad libitum food and water access.

Animal care and the experimental in vivo procedures were performed following the guidelines of the Italian Ministry of Health (Decree Law n. 26/2014), in accordance with the European Directive 2010/63, and all efforts were made to minimize the number of animals used and their suffering. The protocol was approved (Authorization Numbers: 1277/2015-PR and 701/2020-PR) by the Committee set by the Italian Ministry of Health at the National Institute of Health (Rome).

Animals were randomly allocated to the following experimental groups:

(1) MCAo 6 h: mice were subjected to 1 h MCAo followed by 6 h of reperfusion;
(2) SHAM 6 h: sham surgery 6 h before sacrifice;
(3) MCAo 24 h: mice were subjected to 1 h MCAo followed by 24 h of reperfusion;
(4) SHAM 24 h: sham surgery 24 h before sacrifice;
(5) MCAo 48 h: mice were subjected to 1 h MCAo followed by 48 h of reperfusion;
(6) SHAM 48 h: sham surgery 48 h before sacrifice;
(7) MCAo + TSG-6: mice were subjected to 1 h MCAo followed by 48 h of reperfusion and intravenously (i.v.) injected upon reperfusion with 30 μg recombinant mouse TSG-6 (2326-TS, R & D Systems, Minneapolis, MN, USA) dissolved in 100 μL PBS;
(8) MCAo + vehicle: mice were subjected to 1 h MCAo followed by 48 h of reperfusion and i.v. injected upon reperfusion with vehicle (100 μL PBS).

The minimum sample size was evaluated using an a priori power analysis adjusted to obtain a power of 80% at a significance level of 0.05 (OpenEpi software 3.01, Open Source Statistics for Public Health). On the basis of our earlier experience with the MCAo model, we hypothesized a difference in ischemic volume between mice subjected to MCAo and mice exposed to a neuroprotective procedure (i.e., ischemic PC) of about 28 mm^3^ (approximately 30% reduction in infarct size) and a variability (standard deviation) of 15. This led to an estimated minimum sample size of five animals per experimental group.

### 4.2. Surgical Procedure for MCAo in Mice

Focal cerebral ischemia was induced by proximal occlusion of the middle cerebral artery (MCAo) in mice anesthetized with isoflurane as previously described [60,69]. Briefly, vessel occlusion was accomplished by introducing a silicone-coated nylon filament (diameter: 0.23 mm, Doccol Corporation, Redlands, CA, USA) into the internal carotid artery (ICA) for 10–11 mm from its bifurcation from the common carotid artery, whereby a moderate resistance was indicative of proximal MCAo at the level of the Willis circle. In addition to correct positioning of the filament, animals were considered ischemic, and hence included in the study, if presenting >3 of the following deficits assessed after 45 min MCAo: ellipsoidal shape of the palpebral fissure, lateral extension of one or both ears, asymmetric body bending or laterally extending limbs [70]. To allow reperfusion, the filament was withdrawn 1 h after MCAo. Sham mice received the same anaesthetic regimen and surgery as MCAo mice, without introduction of the filament.

### 4.3. Brain Infarct and Neurological Deficits Assessment

To assess cerebral ischemic damage, animals were sacrificed 48 h after the beginning of reperfusion and their brains were dissected and immediately frozen at −20 °C. Fifteen (20 μm thick) coronal slices were cut at 0.5 mm intervals from the frontal pole using a cryostat, then mounted on slides and stained with cresyl violet [71]. Images of cresyl violet-stained sections were captured by a digital scanner and blindly analysed using an image analysis software (ImageJ, version 1.53, NIH, USA) to calculate the infarct volume and oedema [72].

Neurological deficits were assessed 48 h after MCAo or SHAM surgery using the dichotomized De Simoni composite neuroscore to evaluate the general and focal neurological dysfunctions caused by the ischemic insult. Briefly, the total score ranges from 0 (healthy) to 56 (the worst performance in all the 13 categories) and represents the sum of six general deficits (fur (0–2), ears (0–2), eyes (0–4), posture (0–4), spontaneous activity (0–4) and epileptic behaviour (0–12)) and seven focal deficits (body asymmetry (0–4), gait (0–4), climbing (0–4), circling behaviour (0–4), forelimb symmetry (0–4), compulsory circling (0–4) and whisker response (0–4)) [70,71,72,73].

### 4.4. Western Blot Analysis

After 6, 24 or 48 h from MCAo or SHAM surgery, animals were deeply anaesthetized with isoflurane and sacrificed to dissect whole brains or ipsilateral (ischemic) and contralateral frontoparietal cortices (3.2 to −3.8 mm from the Bregma) [74]. Blood was collected in EDTA Vacutainer^®^ tubes (VWR International, Milan, Italy), and plasma was separated by centrifugation at 1500× *g* for 15 min at 4 °C, followed by centrifugation at 16,000× *g* for 15 min at 4 °C to discard the debris and insoluble components.

Brain samples were homogenized in ice-cold RIPA buffer containing protease inhibitor cocktail (PI, Sigma-Aldrich, Milan, Italy) and lysates were centrifuged for 20 min at 20,817× *g* at 4 °C. The supernatants were collected for protein quantification (Bradford protein assay, Bio-Rad Laboratories, Milan, Italy) and an equal amount (30 μg) of proteins was mixed in Laemmli buffer (Sigma-Aldrich, Milan, Italy). Plasma samples were diluted (1:10) in ice-cold RIPA buffer containing PI, and 10 μL of this solution was mixed in 10 μL Laemmli buffer. Samples suspended in Laemmli buffer were loaded onto Mini-PROTEAN^®^ TGX™ Precast Protein Gel for separation in a Mini-PROTEAN Tetra Cell apparatus (Bio-Rad Laboratories, Milan, Italy). Protein gels were next electroblotted using the Trans-Blot Turbo transfer apparatus and a Nitrocellulose Transfer kit (Bio-Rad Laboratories, Milan, Italy). Membranes were rapidly transferred to a blocking buffer (5% non-fat milk in 0.05% Tween-20 TRIS-buffered saline) and incubated with a gentle agitation for 1 h at room temperature. The blots were then incubated overnight at 4 °C, with the following primary antibodies: mouse anti-TSG-6 (1:1000; MABT108, Merck Millipore, Milan, Italy), rabbit anti-Ym1 (1:1000; 60130, StemCell Technologies, Meda, MB, Italy) and mouse anti-β-actin (1:3000; A3853, Sigma-Aldrich, Milan, Italy). This was then followed by incubation with the appropriate secondary antibodies (1:3000; Sigma-Aldrich, Milan, Italy) for 1 h at room temperature [13]. Immunodetection and quantification of protein bands were performed using the iBright™ FL1500 (Thermo Fisher Scientific, Monza, MB, Italy) and ImageJ software.

### 4.5. Real-Time Polymerase Chain Reaction (PCR) in Mouse Plasma Samples

Quantitative real-time PCR analyses were carried out on mouse plasma samples collected (as described above) 6, 24 and 48 h after 1 h MCAo or SHAM surgery. 

MicroRNA was extracted from plasma samples using a miRNeasy Serum/Plasma Kit (Qiagen, Inc., Hilden, Germany). Briefly, 5 volumes of QIAzol lysis reagent were added to plasma and 5 pM *A. Thaliana* miR-159a (478411_mir, Life Technologies, Monza, MB, Italy) was spiked into the mixture [13]. Subsequently, chloroform was mixed in the solution which was then centrifuged for 15 min at 12,000× *g* at 4 °C to obtain three layers. The colourless upper aqueous phase was isolated, mixed with 1.5 volumes of 100% ethanol, transferred into an RNeasy MinElute spin column and centrifuged at 8000× *g* for 15 s at room temperature. The spin column was washed with the supplied wash buffers (RWT and RPE), and then with 80% ethanol. Finally, miRNA was eluted in 14 μL RNase-free water.

According to the manufacturer’s protocol, miRNA quantification was performed using a TaqMan Advanced miRNA Assays Kit (Life Technologies, Monza, MB, Italy) on a QuantStudio™ 3 real-time PCR system (Thermo Fisher Scientific, Monza, MB, Italy). By using the comparative cycle threshold (Ct) method, the relative expression level of *miR-23a-3p* (mmu478532_mir, Life Technologies, Monza, MB, Italy) and *miR-23b-3p* (mmu478602_mir, Life Technologies, Monza, MB, Italy) were calculated by normalization to the expression of *miR-669c-3p* (mmu483332_mir, Life Technologies, Monza, MB, Italy) which remained stable in all the tested samples.

### 4.6. Immunofluorescence

Mouse brains were quickly dissected 24 and 48 h after 1 h MCAo, fixed with paraformaldehyde, cryoprotected in 30% sucrose solution and cryostat-cut into 40 µm-thick coronal sections collected at the level of the regions supplied by the middle cerebral artery (1.18 to −0.10 mm from the Bregma) [74]. Using a previously described method [75,76], colocalization studies were performed on free-floating brain slices by incubating a combination of the following primary antibodies: rabbit polyclonal anti-TNFAIP6 (1:200 dilution; PA599494, Life Technologies, Monza, MB, Italy), rat anti mouse Ly-6B.2 (1:200; clone 7/4; Bio-Rad AbD Serotec, Segrate, Milan, Italy) to label myeloid cells (i.e., granulocytes and monocytes/macrophages), mouse anti-NeuN (anti-neuronal nuclei; 1:200; MAB377, Merck Millipore, Milan, Italy) to label neurons, rabbit anti-platelet and endothelial cell adhesion molecule 1 (PECAM1; 1:200; #5700639; Merck Millipore, Milan, Italy) to label the endothelium. Afterwards, primary antibodies were labelled with the corresponding secondary antibodies conjugated with AlexaFluor 488, AlexaFluor 568 or AlexaFluor 594 (1:200 dilution; Life Technologies, Monza, MB, Italy), while 4′,6-diamidino-2-phenylindole (DAPI, 1:500; Sigma-Aldrich, Milan, Italy) was used to counterstain nuclei. Immunostaining was observed under a confocal laser scanning microscope (Fluoview FV300, Olympus, Segrate, Milan, Italy) equipped with the dedicated software (cellSens v 4.1.1, Olympus) for image analysis.

### 4.7. Ischemic Stroke Patients and Control Subjects

Human blood samples were collected in the frame of a cross-sectional case control study conducted on 25 patients of both sexes with a diagnosis of acute ischemic stroke (within the first 48 h from symptom onset) and 13 age-matched healthy control subjects (CT). Patients were recruited from the U.C. Malattie Cerebrovascolari e Stroke Unit, IRCCS Fondazione Mondino, Pavia, Italy. The study was approved by the local ethics committee (N. p-20170026158) and was conducted following the principles of the Declaration of Helsinki. All patients were assessed for stroke severity and degree of disability using NIHSS and mRS, respectively [77,78]. At the time of enrolment, samples of blood (18 mL) from the cubital vein were collected in sterile tubes from all subjects.

### 4.8. Gene Expression Analysis in Human Peripheral Blood Mononuclear Cells (PBMCs)

PBMCs were isolated immediately after collecting blood samples in EDTA-containing tubes and diluted (1:1) with PBS (Sigma Aldrich, Milan, Italy). Diluted blood samples were slowly loaded into 15 mL Ficoll separating solution (Sigma Aldrich, Milan, Italy) and centrifuged at 800× *g* for 30 min at room temperature. PBMCs, accumulated in the middle white monolayer, were washed twice in sterile PBS at 300× *g* for 15 min and re-suspended in Trizol (Bio-Rad Laboratories, Segrate, Milan, Italy) to be stored at −80 °C until use (up to 2 weeks). The total RNA was extracted from pellets using the Direct-zol RNA Mini prep plus (Zymo Research, Aurogene, Rome, Italy) and the RNA quality was assessed using a spectrophotometer (Nanodrop One/One, Thermo Fisher Scientific, Monza, MB, Italy); cDNA was generated using an iScript cDNA Synthesis Kit (Bio-Rad Laboratories, Segrate, Milan, Italy) following the supplier’s instructions. The gene expression of *TSG-6* was analysed using the Fast Eva Green Supermix (Bio-Rad Laboratories, Segrate, Milan, Italy), using *Ubiquitin C* (*UBC*, whose expression remained constant in all experimental groups) as a housekeeping gene. The primer sequences obtained from the Primers3 software were *TSG-6* forward primer: GTGTGGTGGCGTCTTTACAG, *TSG-6* reverse primer: AGCAACCTGGGTCATCTTCA, *UBC* forward primer: AGAGGCTGATCTTTGCTGGA and *UBC* reverse primer: GTGGACTCTTTCTGGATG. The amplification was performed with a light Cycler 480 Instrument rt-PCR Detection System (Roche, Basel, Switzerland) following the supplier’s instructions. All samples were assayed in triplicate and the gene expression levels were calculated according to the 2 ^− ∆∆Ct^ = 2 − (Ct gene − Ct housekeeping gene) formula by using Ct values.

### 4.9. Statistical Analysis

Data are expressed as means ± S.E.M. for quantitative variables or as medians with interquartile range (IQR) for categorical ordinal variables (i.e., neuroscore) or non-normally distributed data (i.e., *TSG-6* mRNA expression in human PBMCs). Data were subjected to statistical analysis using Graph-Pad Prism software for Windows (version 6.0, GraphPad Software, San Diego, CA, USA). Comparisons between two experimental groups were performed by an unpaired Student’s t test or a Mann–Whitney test. Comparisons between multiple experimental groups were performed using one- or two-way ANOVA followed by Tukey or Bonferroni post-tests, respectively. Values of *p* < 0.05 were considered to be significant.

## Data Availability

Not applicable.
